# Soccer (football) and brain health

**DOI:** 10.1007/s00415-024-12320-5

**Published:** 2024-04-01

**Authors:** Umberto Pensato, Pietro Cortelli

**Affiliations:** 1 Department of Biomedical Sciences, Humanitas University, via Rita Levi Montalcini 4, 20072 Pieve Emanuele, Milan, Italy; 2 IRCCS Humanitas Research Hospital, via Manzoni 56 Rozzano, 20089 Milan, Italy; 3 IRCCS Istituto delle Scienze Neurologiche di Bologna, Bologna, Italy; 4 Department of Biomedical and NeuroMotor Sciences (DIBINEM), University of Bologna, Bologna, Italy

**Keywords:** Sports concussions, Heading, Chronic traumatic encephalopathy, Amyotrophic lateral sclerosis, Neurological disorders, Head trauma

## Abstract

Soccer is one of the most popular sports worldwide, played by over 270 million people and followed by many more. Several brain health benefits are promoted by practising soccer and physical exercise at large, which helps contrast the cognitive decline associated with ageing by enhancing neurogenesis processes. However, sport-related concussions have been increasingly recognised as a pressing public health concern, not only due to their acute impact but also, more importantly, due to mounting evidence indicating an elevated risk for the development of neurological sequelae following recurrent head traumas, especially chronic traumatic encephalopathy (CTE). While soccer players experience less frequent concussions compared with other contact or combat sports, such as American football or boxing, it stands alone in its purposeful use of the head to hit the ball (headings), setting its players apart as the only athletes exposed to intentional, sub-concussive head impacts. Additionally, an association between soccer and amyotrophic lateral sclerosis has been consistently observed, suggesting a potential “soccer-specific” risk factor. In this review, we discuss the neurological sequelae related to soccer playing, the emerging evidence of a detrimental effect related to recurrent headings, and the need for implementation of comprehensive strategies aimed at preventing and managing the burden of head impact in soccer.

## Introduction

Soccer is one of the most popular sports worldwide, played by over 270 million people and followed by many more [1]. Numerous aspects of human health are positively influenced by soccer and physical activity in general, including brain health through the promotion of neurogenesis [2–4]. Nonetheless, soccer, like all sports that expose athletes to repetitive head impacts, may result in acute brain damage and also neurological sequelae. Notably, chronic traumatic encephalopathy, a neurodegenerative disease that affects specifically athletes exposed to repetitive head injuries [5], has been recently linked also to soccer [6]. Indeed, even though the head impact exposure is likely less relevant in soccer than in other contact or combat sports, such as American football or boxing, the former is characterised by a distinct move, the heading, whose effects on the CNS have been studied only over the last few years [7]. Additionally, a controversial, yet worrisome correlation between amyotrophic lateral sclerosis and soccer has recently emerged, which seems to be soccer specific, leading to the hypothesis that head trauma may not be the main putative factor. In this review, we will delve into the nuanced beneficial and detrimental impact of soccer playing on brain health, with particular emphasis on concussion sequelae, chronic traumatic encephalopathy, and amyotrophic lateral sclerosis.

## Search strategy and selection criteria

We used the following terms with no language restrictions to search PubMed from starting date to 31 December 2023, and we reviewed all titles: (soccer OR football [title/abstract]) AND (neurologic* OR traum* OR neurodegeneration OR brain OR concussion [title/abstract]). We then identified and screened 3432 titles. We used tags to identify relevant papers, including “soccer”, “traumatic chronic encephalopathy”, “concussion”, “headings”, and “amyotrophic lateral sclerosis”. We prioritised papers in the last 20 years for this review.

## Neuroprotective effects of soccer

Running and physical activity play a critical role in promoting overall body and brain health throughout life, helping prevent and mitigate brain damage and ageing [2–4]. Even though no empirical study has directly probed the brain health advantages of soccer in human subjects, the neuroprotective benefits extrapolated from extensive research on aerobic exercise and running activities can arguably be extended to soccer playing. Exercise benefits may be attributed to promoted neurogenesis and enhanced brain blood flow [2, 3, 8]. This beneficial impact on brain health is deeply rooted in human evolution, linking physical activity to survival and providing an evolutionary advantage to active human beings [9].

The benefits of physical exercise on the brain during adolescence may have a more lasting effect compared to later stages. This is likely due to the morphological and functional reorganisation that occurs during this period when there is higher neuroplasticity, promoting lasting changes in response to environmental stimuli, including exercise [10].

Ageing is marked by significant brain structure and function alterations, resulting in cognitive decline [11, 12]. These changes are primarily driven by inflammatory responses, oxidative stress, and diminished synaptic plasticity. This culminates in the permanent loss of neurons and white matter volume, alongside a gradual decline in neurogenesis, particularly evident in the hippocampi [13]. Physical activity has been shown to reduce the rate of decline in older people [14]. Indeed, aerobic exercise decreases the impact of atherosclerotic cerebrovascular disorder and is linked to larger hippocampal volume in healthy older individuals [2, 13]. Regular exercise in old age can increase hippocampal volume by up to 2% and enhance performance in memory and attention tasks [15–17]. Physical activity is associated with a lower risk of Alzheimer’s disease (AD) [18] and improved survival rates in AD patients [19]. Regular exercise has been shown to reduce amyloid plaque deposition, preserve brain volume, and decrease temporal lobe atrophy [20]. Emerging evidence suggests that physical activity is also linked with a substantial decrease in the likelihood of developing Parkinson’s disease (PD) [21–23] and slows disease progression [24]. Collectively, these data support a direct effect of exercise on neurodegeneration prevention, yet a few limitations should be acknowledged. The reduced exercise levels observed in individuals who subsequently manifest neurodegenerative disorders may potentially stem from either motor or cognitive subclinical manifestations of the disorders in their preclinical stages. Moreover, exercise may indirectly exert its preventive effects on neurodegeneration by increasing the motor and cognitive reserve—the capacity to maintain normal neurological function despite underlying damage—the brain resilience [25]. Accordingly, a recent groundbreaking study found that a healthy lifestyle in older individuals, including regular physical activity, is associated with improved cognitive function [26]. Remarkably, this association persists independently of common neurodegenerative pathology modifications [26], substantiating the concept that a large contribution of physical activity’s role in preventing neurodegenerative disorders might be mediated by increased cognitive reserve.

## Soccer-related head trauma

Contact sports athletes are frequently subjected to a multitude of head injuries throughout their athletic endeavours. Sport-related concussions have been increasingly recognised as a pressing public health concern, not only due to their acute impact but also, more importantly, due to mounting evidence indicating an elevated risk for the development of neurological sequelae following recurrent head traumas [27, 28]. Remarkably, the sport of soccer, despite its emphasis on finesse and skill over physicality, presents a notable risk of enduring repetitive head injuries, irrespective of the player’s position on the field. For instance, in the prestigious 2014 FIFA World Cup, the second most injured body part was the head, accounting for 18% of all traumas [29].

**Fig. 1:**
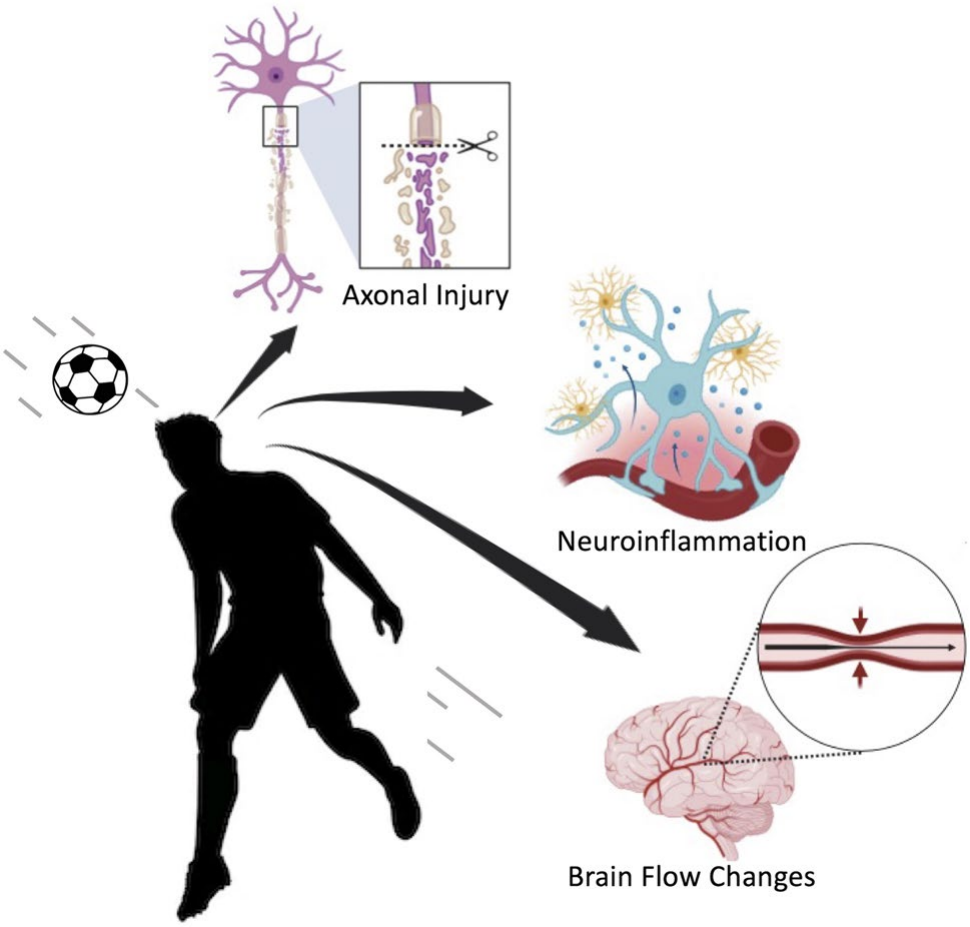
Pathological changes associated with concussions and sub-concussions (headings)

Specifically, soccer players are exposed to two different types of head impacts, stemming from both “unintentional” head trauma and the deliberate act of heading the ball. Notably, in contrast to other sports, soccer stands alone in its purposeful use of the head to hit the ball, setting its players apart as the only athletes exposed to “intentional” head impacts. It has been estimated that a typical soccer player executes an average of 6–12 headers per game and engages in a minimum of 2000 headers over a 20-year professional career, alongside countless repetitive heading drills during training sessions [7]. While singular headings do not directly lead to neurological symptoms or concussions, they contribute significantly to the accumulation of sub-concussive impacts, which have been linked to potential cognitive function impairment [30–33] (Fig. 1).

### Sub-concussion, concussion, traumatic brain injury, and neurological sequelae

The vast majority of individuals exposed to head impacts typically experience trauma confined to the cranial structure without significant involvement of the brain parenchyma. Instead, a minority of subjects experience the development of loss of consciousness or the onset/worsening of neurological signs or cognitive impairment following a head trauma—traumatic brain injury (TBI) [34]. The pathophysiology of such injuries involves functional damage that often eludes detection by conventional neuroimaging, such as CT or MRI, primarily designed to identify structural damage [35, 36]. TBIs are commonly graded at the onset based on the Glasgow Coma Scale (GCS) into mild (GCS 13–15), moderate (GCS 9–12), and severe (GCS < 9). While only severe TBIs are usually linked to evident structural damage, it is noteworthy that even mild or moderate TBIs can infrequently manifest with intracranial lesions [34, 36].

Many authors utilise the term concussion to describe any neurological manifestation following head trauma in the absence of discernible intracranial lesions on neuroimaging, suggesting that the term can be used interchangeably with mild to moderate TBI most of the time [35, 37]. On the other hand, a patient may incur a brain injury even in the absence of overt symptoms, as the nature of the trauma may have been too subtle to generate symptoms or be discerned through clinical assessment. In such instances, the term “sub-concussion” is employed to characterise these covert injuries [38].

Both isolated TBI and repetitive TBI or sub-concussions can elicit a spectrum of diverse neurological sequelae (Table 1) [39–41]. Common persistent symptoms include headache, dizziness, impaired attention, poor memory, executive dysfunctions, irritability, and depression [42]. Post-concussion, persistent post-concussion, and chronic post-concussion syndrome have an acute onset temporally related to a single concussive event, whereas chronic traumatic encephalopathy (CTE) occurs insidiously following multiple head injuries and a latent period.

**Table 1:** Neurological sequelae of sport-related head trauma

	Onset and progression	Type of trauma
Post-concussion syndrome	Acute/subacute onset (usually resolved within 10 days)	Isolated head trauma (usually mild to moderate)
Persistent post-concussion syndrome	Acute/subacute onset (persisting more than 30 days, possibly reversible)	Isolated head trauma (usually moderate to severe)
Chronic post-concussion syndrome	Acute/subacute onset (persisting more than 1 year, unlikely reversible)	Isolated head trauma (usually moderate to severe)*
Post-traumatic dementia or parkinsonism **	Acute/subacute onset (irreversible)	Isolated head trauma (usually moderate to severe)*
Chronic traumatic encephalopathy (CTE)	Insidious onset and progressive course	Repetitive concussive and sub-concussive blows

*Athletes may have a history of previous multiple TBI

**Other neurological sequelae, such as motor symptoms, are possible

TBI: Traumatic Brain Injury

### Soccer-related acute head trauma assessment and management

The acute assessment of sport-related head trauma in soccer, similar to other sports, emphasises early recognition, especially of moderate-severe TBI, that needs to be addressed in a hospital setting to perform neuroimaging evaluations [37]. Sub-concussive events are not addressed in this section due to current recognition limitations. A critical part of the side-line assessment is the rapid screening for suspected concussion rather than making a definitive diagnosis. Players with suspected concussion following a significant head impact or displaying symptoms should undergo a side-line screening using appropriate assessment tools. The side-line assessment should quickly evaluate consciousness, cranial nerves, and balance while identifying potential severe injury warning signs. The Sport Concussion Assessment Tool (SCAT) 6 and child SCAT6 are the most established instruments for side-line assessment [37, 43]. Delayed-onset symptoms of concussion are well documented, underscoring the importance of follow-up evaluations after a suspected concussion, even if initial side-line screening is negative. If SCAT6 indicates a potential concussion, the athlete should be promptly removed from playing. Despite being well established, these recommendations are often largely disregarded [37, 44]. The SCAT protocol’s requirement to isolate the affected athlete for 10–15 min may contribute to its low compliance. While feasible in American Football due to the possibility to pause game time, in soccer, where the game continues without interruption, athletes are often eager to resume play, leading to an increased risk of undetected concussions.

After removal from play, a resting phase is recommended to mitigate post-concussion symptoms [45]. This rest period also helps reduce brain energy demands following a concussion, promoting recovery. A gradual return-to-play strategy should be implemented after the initial resting phase (24–48 h). A sufficient period of absence from sports participation protects athletes from the harmful effects of repeated concussions. Clinical recovery involves the complete resolution of post-concussion-related symptoms, usually occurring in 7–10 days for most athletes. Persisting symptoms (> 4 weeks) should be evaluated with multimodal clinical assessment and can benefit from rehabilitative interventions [37]. Immediate symptom burden represents the most important prognostic factor for prolonged recovery and risk of developing chronic post-concussion syndrome [46–48]. Other potential contributors shared with other sports include younger age and prior history of concussion, headache, and psychiatric issues [46, 47, 49, 50]. Notably, the intricate process of brain recovery may extend beyond the period of clinical symptoms, potentially exposing the athletes to heightened risks if they prematurely return to play [45].

### Soccer-related head trauma prevention

No proven treatment currently exists for the long-term effects of head trauma; hence, prevention is critical. Limiting athletes’ exposure to concussive and sub-concussive head impacts is the primary approach, involving different strategies to reduce the frequency and intensity of traumas (Table 2) [37, 51].

**Table 2:** Strategies to reduce the risk of neurological complication due to head impacts

Reduced frequency of head impacts	- Soccer policy or rule changes
Reduced intensity of head impacts	- Headgear - Neck muscle strength - Properties of the ball and playground surface
Early recognition of concussion	- Reduce barriers - Education - Spotters - Biomechanical sensors

First, the employment of stricter penalties, such as suspension for multiple matches for players causing avoidable head trauma to opponents, can be introduced [6]. Indeed, studies showed a decrease in head-to-head and elbow-to-head incidents when referees were given the authority to penalise intentional elbow-to-head contact with immediate red cards [52, 53].

Second, soft-shelled headgear equipped with energy-absorbent padding has been shown to mitigate the intensity of head trauma during simulated head-to-head impacts [54–56].

However, its results in heading have been controversial [57] and its applicability in soccer, where heading plays a crucial role, presents a significant challenge. Furthermore, the introduction of headgear may alter player’s behaviour by instilling a misguided sense of protection, potentially leading to more aggressive play [58].

Third, strengthening the neck muscles can minimise head acceleration, reducing the risk of head injuries [59]. Notably, neck muscles are receptive to short-term training, as demonstrated in professional rugby players [59, 60]. However, the effectiveness of neck strengthening on headings is still controversial [61].

Fourth, it is essential to consider the characteristics of the ball and the playing surface in the context of head injury prevention. The former might be particularly important for young players who should play with an appropriately sized ball and attend to the inflation pressure of the ball [62, 63].

Fifth, an integral component of any effective concussion management programme lies in the prompt detection of potential injuries. The key to facilitating injury identification hinges on educating not only referee, who holds the authority to halt the game, but also other figures, such as athletes, athletic trainers, sports medicine physicians, and coaches, who can alert the referee [50].

Sixth, the employment of in-game spotters who actively assess live gameplay represents a valuable asset in identifying concealed head injuries [64]. This strategy has been explored in the 2018 FIFA World Cup in Russia; yet, implementing such programmes is complex and cost-intensive, making them inaccessible for most non-elite organisations [50].

Seventh, biomechanical sensors, applicable to helmeted and non-helmeted sports (e.g. mouthguard or earpiece), are a promising avenue. These sensors operate akin to radiation sensors used by radiologists, triggering alerts when a hazardous threshold is surpassed. However, they have not demonstrated a distinct “concussion threshold”—the magnitude of impact alone proves insufficient for predicting concussion—and have not yet undergone validation for use in soccer [65].

Finally, obstacles intrinsic to soccer games hinder the prompt detection and management of head injuries, including a rapid pace, extensive playing area, substitution regulations, absence of time-outs, and the highly competitive nature that renders players reluctant to report symptoms due to the apprehension of being side-lined.

### The National Football League concussion protocol

The National Football League (NFL) has implemented a comprehensive protocol to address the prevention, detection, and management of concussions in American football [66–68]. During the preseason and offseason, players undergo education on concussions and a club-appointed neuropsychologist administers baseline neurocognitive tests, which are then stored in a centralised electronic medical record system for the league. Before every NFL game, a 60-min medical team meeting reviews protocols and roles. During games, the athletic trainer spotters use video records to assist the medical staff in identifying concussions. If a suspected concussion occurs, a standardised examination protocol is followed to detect signs that prevent the players from continuing. Additionally, a return-to-participation protocol after a concussion has also been established. Notably, the NFL concussion protocol aligns with SCAT recommendations, but it is tailored for a specific sport—American football—and a specific league—the NFL [37]. This comprehensive protocol may offer valuable strategies for other sports, including soccer.

## Chronic traumatic encephalopathy

Chronic traumatic encephalopathy (CTE) represents a progressive neurodegenerative condition attributed to repetitive concussive or sub-concussive head trauma [41]. Notably, its neuropathological features stand apart from other neurodegenerative disorders, manifesting as the perivascular accumulation of phosphorylated tau in neurons and astrocytes, specifically concentrated within the sulci [41].

This condition was first described in 1926 when it emerged among retired boxers, manifesting as a constellation of behavioural and cognitive symptoms. Therefore, it was initially named “punch-drunk syndrome” [69] or “dementia pugilistica” [70], subsequently evolving into CTE [41]. Later, the same manifestations were also observed in numerous athletes involved in not only other contact sports, prominently American football [71], but also soccer [72]. The very first documented case of CTE in a soccer player unfolds as a captivating narrative centred around Bellini, the iconic captain who led the Brazilian soccer team to be victorious in Brazil’s inaugural Football World Cup in 1958 and who was honoured with a statue outside the Estadio do Maracana in Rio de Janeiro. He was initially diagnosed with Alzheimer’s disease, yet a post-mortem neuropathological examination revealed findings consistent with CTE [73]. His affliction with CTE remained shrouded in obscurity until 2014 when his story was published in the New York Times [74] and later in a book written by his wife—“Bellini: The First Captain Champion”. In the following years, his case catalysed a growing awareness of the condition, leading to the emergence of additional pathologically confirmed CTE cases among retired soccer players [6, 33, 72, 75].

The precise epidemiology of CTE among retired soccer elite athletes remains uncertain, yet a remarkable anatomopathological investigation revealed an astonishing prevalence of CTE at 87% in a cohort of 202 deceased individuals with a history of repetitive head trauma related to either contact sport or military service [71].

Notably, recurrent concussion or sub-concussion episodes represent a necessary but not sufficient factor to develop CTE. Indeed, the pathogenesis of CTE is influenced by a multifaceted interplay of genetic, environmental and behavioural factors, collectively determining the individual cumulative “head injury threshold” necessary for its manifestations. Risk factors include age, length of soccer career, time elapsed since retirement, frequency of concussions/sub-concussions, and the presence of the apolipoprotein ε4 genotype [76, 77].

### Clinical manifestations

CTE usually presents as progressive cognitive, behavioural, and mood changes [5, 41]. Cognitive impairment, prominently affecting attention, executive functions, and memory, often predominates in the initial clinical presentation [78]. Accompanying behavioural disturbances manifests as depression and heightened aggression, including emotional volatility, physical and verbal confrontations, and impulse dysregulation [78]. These symptoms can result in significant personal challenges, such as substance misuse, financial instability, and interpersonal discord. Motor symptoms are present in a subgroup of patients at presentation and are usually subtle. Nonetheless, meticulous clinical assessments remain pivotal as motor manifestations such as motor neuron disease, parkinsonism, and cerebellar dysfunction have been recurrently documented [41].

### Diagnosis

**Fig. 2:**
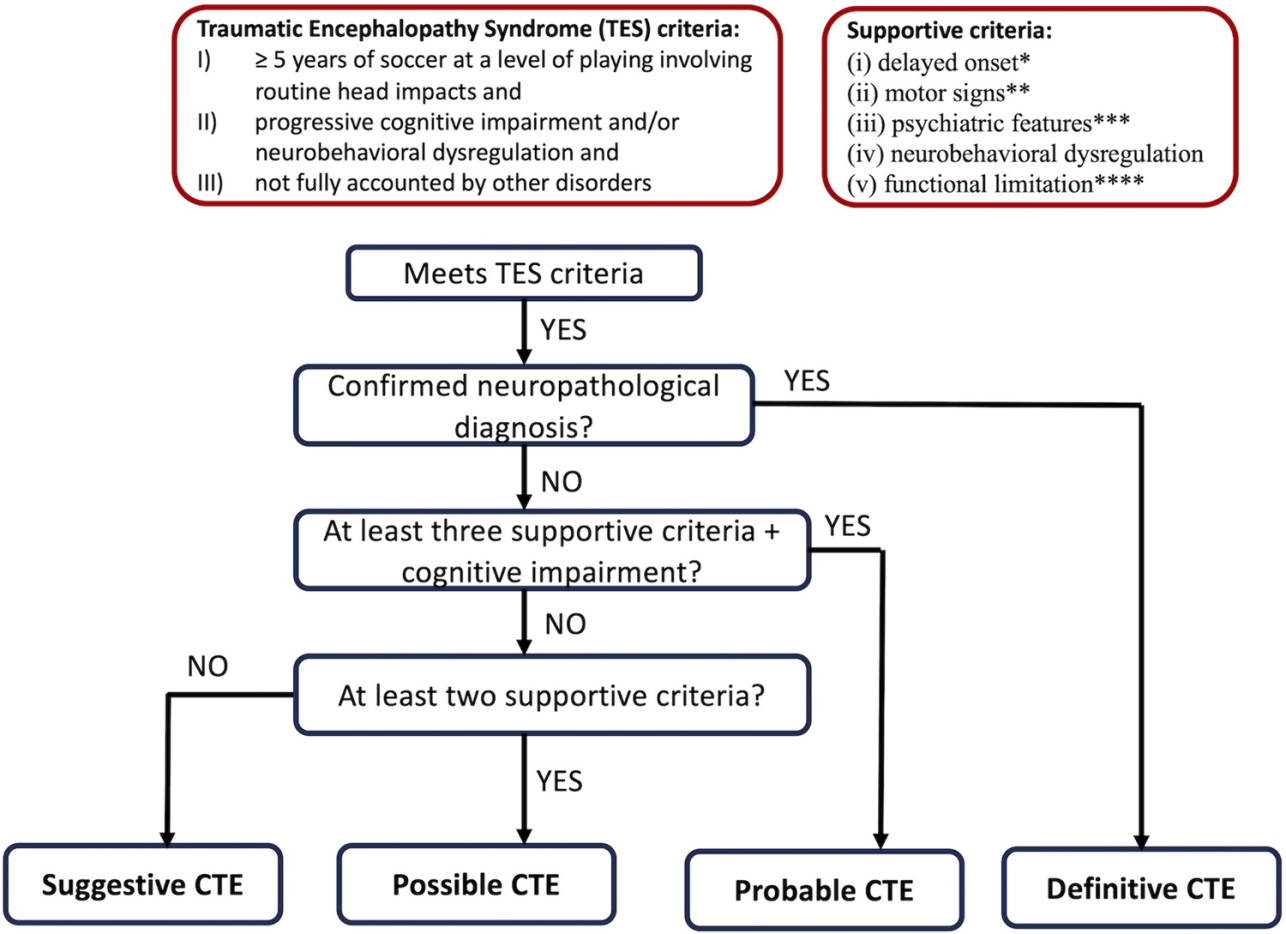
Flow diagram for determining Chronic Traumatic Encephalopathy (CTE) research diagnostic criteria in soccer players. * The minimum period of clinical stability after head impact exposure has not been defined but should be several years. ** Parkinsonism, motor neuron disease, or others *** Anxiety, apathy, depression, or paranoia. **** Functional limitations should at least reflect subtle functional limitations for possible CTE and mild dementia for probable CTE

As in other neurodegenerative disorders, neuropathological assessment stands as the gold standard for a diagnosis of CTE, rendering a definitive diagnosis in living individuals unattainable [79]. Currently, only research diagnostic criteria have been validated (Fig. 2) [79, 80]. Within these criteria, the term traumatic encephalopathy syndrome (TES) has been employed to characterise the clinical phenotype linked to repetitive head impacts. Several distinctive structural and functional neuroradiological features can provide valuable support for the diagnosis of CTE [41, 81, 82], even though they have not been included in the last research diagnostic criteria due to a lack of robust validity [79]. Structural brain features include cortical thinning, atrophy, and the presence of a cavum septum pellucidum [41, 82]. The latter is explained by the capacity of fluid waves in the context of trauma to induce fenestrations within the septum, facilitating fluid entry between the leaflets and resulting in separation within the structures. Yet, this radiological sign can be observed in up to 15% of healthy individuals [83]. Regarding functional neuroimaging, positron emission tomography (PET) and single-photon emission computed tomography (SPECT) have emerged as promising tools for CTE diagnosis. These imaging modalities have shown glucose hypometabolism and hypoperfusion patterns in key brain regions, including the frontal-parietal-occipital lobes, posterior cingulate cortex and cerebellum [84, 85]. Moreover, groundbreaking studies leveraged cutting-edge imaging techniques such as flortaucipir PET and florbetapir PET to assess the deposition of tau and amyloid beta proteins in the brains of living CTE patients, unveiling a distinctive signature characterised by elevated levels of tau deposition alongside normal amyloid beta levels—especially in bilateral superior frontal, bilateral mesial temporal, and left parietal lobe [81]. Remarkably, a consistent molecular signature characterised by elevated Tau levels in parallel with low amyloid beta levels has also been demonstrated in the cerebrospinal fluid of CTE patients [86].

### Therapy

Regrettably, no disease-modifying intervention is currently available for CTE, relegating clinical management to supportive care. Additionally, no therapeutic clinical trial has been conducted so far in CTE. Nonetheless, the same classes of medications employed in other neurodegenerative diseases can be cautiously attempted in CTE patients [41].

## Amyotrophic lateral sclerosis

Amyotrophic lateral sclerosis (ALS) is a neurodegenerative disorder characterised by the progressive degeneration of both lower and upper motor neurons, leading to a relentless fatal trajectory [87]. The estimated prevalence is 5 per 100.000 people with an average age of onset of 56 years and 46 years for sporadic and familial cases, respectively [88, 89]. The spinal form is the most common, yet 20–30% present with bulbar onset, and an even smaller subgroup may present with respiratory symptoms or frontotemporal dementia [88, 89]. Despite a limited understanding of its biological underpinnings, various putative pathogenic mechanisms have been proposed, including excitotoxicity, oxidative stress, damage to axonal transport, and protein aggregation [88, 89].

ALS has been historically also known as Lou Gehrig’s disease, after the legendary New York Yankees baseball player who died from this disease at the young age of 38 years old [87], sowing doubts for the first time about the potential relationship between elite sports and the disorder.

Subsequently, a notable elevation of 6.5-fold increase in the likelihood of developing ALS has been documented within a large cohort of 7325 elite soccer players in Italy – 8 observed cases versus 1.24 expected occurrence [90]. Moreover, these eight patients exhibited distinctive features, including a high incidence of bulbar onset, a predominance of midfielders, and an early age of onset. The authors also explore the ALS prevalence in basketball players and cyclists cohorts, both sharing potential risk factors with soccer, such as intense physical activity and risk of illegal drug use; yet, no ALS cases were found, ultimately suggesting an unveiled soccer-specific risk factor [90]. Other Italian studies confirmed an increased risk of ALS in former soccer players, substantiating their pathogenic relationship [91, 92]. This potential link has gained significant interest in Italian neurologists after the investigation of a prosecutor into the premature deaths from ALS of several former soccer athletes, prompted by concerns about illicit drug use. While sporadic reports of ALS in soccer players have emerged in other European countries, the correlation between professional soccer and ALS has not been thoroughly explored beyond Italy. While the roots underlying this relationship have not been untangled, different possible soccer-related risk factors have been proposed.

### Head traumas and ALS

The theory proposing a potential link between ALS and antecedent head trauma was born as early as the 1910s, inspired by observations suggesting that ALS symptoms sometimes emerge after traumatic events. As further investigations revealed that overall trauma rates did not significantly precede the onset of ALS, research attention moved towards head trauma [93].

In support of this hypothesis, an apparent excess of ALS cases was found not only in soccer players but also in US footballers [94], in whom a risk factor as high as 40-fold was found in a retrospective study [95], and not among other athletes such as road cyclists and basketball players who do not share exposure to head injury [90]. Mounting evidence revealed a noteworthy association between individuals who experienced multiple head injuries and the risk of ALS [96, 97]. This has led to a proposal by some authors that ALS in soccer players might be envisioned as a subtype of CTE, yet it remains a controversial hypothesis [98].

### Exercise and ALS

The impact of heightened physical exertion on ALS has been a subject of debate. Oxidative stress and glutamate excitotoxicity might serve as key drivers behind the potential repercussions of excessive physical activity. Recent studies and meta-analyses have demonstrated a robust correlation between a history of physical activity and the risk of developing ALS [99–101], especially in patients with a susceptible genotype [102]. Nonetheless, the level of correlation observed was consistently modest and it is essential to acknowledge the potential influence of confounding factors, such as trauma exposure, which may have contributed to the results. Therefore, all these findings must be interpreted cautiously. While the intuitive connection between vigorous physical activity and the onset of ALS is rooted in the disorder’s well-established association with neuronal excitotoxicity, further evidence is warranted to draw definitive conclusions on this relationship.

### Substance overuse and ALS

Drug abuse, including both legitimate pharmaceuticals and illicit substances, has also emerged as a compelling risk factor for ALS. A minority of professional soccer athletes exhibit a propensity for excessive consumption of medications and nutritional supplements, while a notable prevalence of anti-inflammatory drug use has been observed among individuals afflicted by ALS compared to the general population [103]. However, a comprehensive study involving a cohort of 780.000 individuals, among whom 708 subsequently developed ALS, failed to reveal any discernible relationship between anti-inflammatory drug use and the development of this condition [104].

### Dietary supplements and ALS

Branched-chain amino acids (BCAAs), popular among athletes for muscle growth and recovery, have been suggested to elevate ALS risk in professional soccer and American football players [90, 105, 106]. Notably, in a multicentre Italian trial, excess mortality in patients treated with BCAAs has been observed [107], while in a US trial BCAAs were associated with pulmonary function worsening [108]. High doses of BCAAs over an extended period led to increased excitability in cultured cortical neurons and pyramidal neurons from the motor cortex slices of mice, suggesting a shared phenotype between neurons treated with BCAAs and neurons from genetic ALS mouse models [109]. However, a recent study investigating pre-diagnostic plasma levels of BCAAs from five extensive cohort studies did not find any association between these compounds and the risk of ALS [110].

### Exposure to pesticides and ALS

A distinctive characteristic of soccer players that somewhat sets them apart from athletes in other sports is their frequent and prolonged interaction with natural grass surfaces, similar to farmers. This connection is significant as farmers have been identified as a population group with an elevated risk of developing ALS [111]. The utilisation of pesticides in agricultural practices has been extensively studied and implicated in the pathogenesis of ALS, with compelling evidence suggesting potential dose–response relationships between pesticide exposure and the development of this debilitating condition [112–114]. Nonetheless, no clear association was found between ALS and gene polymorphism of paraoxonase, an enzyme crucial for detoxifying organophosphate pesticides [115].

## Conclusions

Soccer is a captivating and dynamic sport that, like every aerobic physical activity, promotes several benefits for brain health, contrasting the cognitive decline associated with the natural process of ageing. Nonetheless, sport-related concussions may result in several neurological sequelae, including insidious CTE. Regarding the link between soccer and ALS, the evidence remains inconclusive so far, necessitating further exploration in larger studies, especially beyond Italy. Notably, the “soccer-specific trigger” source remains elusive, possibly reflecting its multifactorial nature.

Therefore, it cannot be overstated the need for the development and implementation of comprehensive strategies aimed at both preventing and managing the burden of head impacts in soccer. Notably, the emerging detrimental effect associated with the accumulation of headers represents a distinctive challenge that demands further exploration and scrutiny.

While soccer athletes constitute a minority within the global landscape of CTE and ALS, delving into this unique cohort may hold promise to unveil the pathogenic underpinnings of these devastating neurodegenerative disorders.

## Data Availability

Data sharing is not applicable to this article as no new data were created or analyzed in this study.
